# At Last! Universal Health Coverage That Prioritizes Health Impact: The Latest Edition of Disease Control Priorities (DCP3)

**DOI:** 10.9745/GHSP-D-18-00193

**Published:** 2018-06-27

**Authors:** James D. Shelton

**Affiliations:** a Editor-in-Chief Emeritus, Global Health: Science and Practice, Baltimore, MD, USA.

## Abstract

Sadly, we face a vast sea of health problems in global health. Universal health coverage programming should prioritize interventions with the most health impact, but instead largely succumbs to emphasizing less impactful clinical curative services. In contrast, DCP3 provides an evidence-based template that prioritizes impact. Yet even the most basic and realistic DCP3 package comes at a formidable price.

See related article by Paul.

*Faced by the vast numbers of health problems of mankind, one immediately becomes aware that all of them cannot be attacked simultaneously.*

- Julia Walsh and Kenneth Warren, 1979^1^

## HEALTH OR HEALTH SERVICES?

Universal health coverage (UHC) has become a major focus of global health, embedded in Sustainable Development Goal (SDG) 3 (ensure healthy lives and promote well-being) and a major priority for the World Health Organization (WHO). Thus, according to WHO^2^:

*UHC means that all individuals and communities receive the health services they need without suffering financial hardship. It includes the full spectrum of essential, quality health services from health promotion to prevention, treatment, rehabilitation, and palliative care.*

Certainly, this objective sounds laudable. But notice that health impact is not explicit in the definition. Rather, emphasis is indirect, on a potential *means* to better health—“health services.” And only by mention of the phrase “they need” is impact implied.

Of course, “services” can encompass any health intervention. But many highly effective health interventions such as tobacco taxation, community distribution of long-acting insecticide-treated nets, clean air regulation, and promotion of healthy lifestyle lie outside the clinical realm entirely. And conceptually most people don't tend to think of them as “health services.” Moreover, many high-priority preventive clinical services such as immunization, contraception, and prenatal care can be lost in the shuffle within the vast panoply of curative services.

## THE INEXORABLE PULL TOWARD CURATIVE SERVICE

To most of the public and to politicians “health coverage” means having a doctor or other health care provider available when they need or want one, which generally means after some problem develops. And policy decisions are heavily influenced by physicians, whose main professional orientation is curative practice. Elite decision makers themselves may have personal felt needs for specialized clinical care. Moreover, nascent health insurance systems, which often serve as a core part of UHC, are oriented toward specific, mainly curative, procedures. Thus, UHC, as it has been commonly rolled out, has a primary emphasis on curative clinical, sometimes even highly specialized, care.^3^^,^^4^ And it quickly gets enmeshed in the dense quagmire of issues related to coverage and payment schemes. Yet, ironically, high expenditures on clinical service show scant effect on population-level health indicators.^5^

## ENTER THE DCP'S LATEST EDITION, WITH EVIDENCE-BASED PRIORITY UHC PACKAGES

The prestigious pedigree of the *Disease Control Priorities* (DCP) extends back for decades, convening world-class expertise and using the best available evidence to do exactly what its name conveys—provide guidance on priorities for health programming. After years in the making, its third edition (DCP3) has now blossomed forth.^6^ It aims directly at UHC priorities, using solid criteria of (1) value for money, (2) burden addressed, and (3) implementation feasibility.

### DCP3's Adept Methodology

As befits the complex issue of UHC, the methodology is thoughtfully complex. It starts by laying out interventions like tobacco tax that lie outside the conventional concept of a health system, with 71 “intersectoral” interventions including 29 proposed for early adoption. Then, within the conventional health system, it includes 218 health interventions labeled Essential Universal Health Coverage (EUHC), of which a subset of 71 are included in a Highest Priority Package (HPP). In essence, the HPP is aimed at lower-income countries (LICs) and the larger EUHC at lower-middle-income countries (LMICs).

In addition to looking at interventions individually, DCP3 examines 18 “cluster packages” (such as child health and tuberculosis) as well as 5 “platforms” (such as population, community, and first-level hospital). Interventions are also categorized as urgent, chronic, and time-bound but not urgent. Lastly, the interventions within the EUHC and the HPP subset are evaluated for cost and health impact in the context of the clusters and platforms. (The intersectoral interventions are *not* included in this component of the analysis.)

### The Packages Are Well Prioritized. Yet Very Much Is Not Included Even in EUHC

To help you grasp a sense of what is included or not in the packages, Box 1 shows some examples of what interventions are included in the packages. And Box 2 shows revealing examples that are not. (In some cases, I've condensed and combined categories for simplification.)

- **Intersectoral set and HPP (Box 1).** Many of these interventions will be familiar to those working in global health, featuring interventions (often preventive in nature) well known to be effective—for example, tobacco tax in the intersectoral group and childhood vaccination in the HPP. A modest modicum of fairly basic clinical curative and palliative services are also included in the HPP such as management of childhood illness.
- **EUHC interventions, beyond those in the HPP (Box 1).** Included here are a number of important but less cost-effective preventive interventions such as flu vaccine for high-risk individuals and a larger list of clinical services like suturing of lacerations and limited approach to hypertension.
- **Much not included, even in EUHC (Box 2).** Perhaps most revealing, this list demonstrates the vast expanse of the health arena that such a practical essential package does *not* include. Virtually everyone reading this list will see several, and probably multiple, interventions or conditions for which they themselves or close friends and family have sought care. Notably with respect to the huge and growing burden of chronic disease, treatment of cancer in the EUHC is limited to just early treatment for cervical, breast, and colorectal cancer, leaving the many other cancers, from leukemia to brain cancer, untouched. Likewise most advanced therapy for other major chronic diseases like myocardial infarction, obstructive pulmonary disease, stroke, and heart failure is excluded.

BOX 1 **Illustrative Examples of Health Interventions Included in DCP3**

**Intersectoral Interventions for Health**

- Outdoor air pollution restriction (transport, power, industrial)
- Tax on tobacco, alcohol, and other addictive substances
- Ban on trans fat
- Reduced salt in food products
- Traffic calming mechanisms in road construction
- Infrastructure conducive to walking, cycling

**HPP Interventions**

- Mass media promotion of healthy eating and physical activity
- Routine childhood vaccination
- Contraception
- Labor and delivery (multiple levels)
- Rapid tests and ACTs for malaria
- Management of childhood illness (iCCM and IMCI)
- Management of severe childhood infections
- TB diagnosis and treatment (multiple levels)
- Mass treatment of NTDs (e.g., lymphatic filariasis)
- Condoms for key at-risk populations
- Antiretroviral therapy for HIV
- Postabortion care
- Basic management of depression
- Drainage of superficial abscess
- Adult febrile illness, management (multiple levels)
- Diabetes among adults, basic management
- Selected cardiovascular disease, basic management
- Pain management and palliative care (multiple levels)
- Diagnosis and treatment of early cervical, breast, and colorectal cancer
- Hernia repair
- Cleft palate repair
- Cataract removal and replacement
- Selected urgent surgery (e.g., appendectomy, perforated ulcer)

**Other EUHC Interventions Not in the HPP**

- Mass media to reduce tobacco and alcohol use
- Breastfeeding promotion by lay health workers
- Life skills education
- Hepatitis B vaccination for high-risk adults
- Insecticide-treated nets for children and pregnant women
- Preexposure prophylaxis with antiretroviral therapy for high HIV risk
- Flu vaccine for those with underlying lung disease
- Suturing of lacerations
- Hypertension, opportunistic screening and basic treatment for high risk
- Asthma and obstructive pulmonary disease, simple and some advanced treatment
- Dental caries and extraction
- Gallbladder removal
- Prosthetics, orthotics, and splints
- Repair club feet
- Specialized TB care (e.g., treatment of drug-resistant TB failure)

Abbreviations: ACTs, artemisinin-based combination therapies; DCP3, Disease Control Priorities, 3rd edition; EUHC, Essential Universal Health Coverage; HPP, Highest Priority Package; iCCM, integrated community case management; IMCI, integrated management of childhood illness; NTDs, neglected tropical diseases; TB, tuberculosis.

BOX 2 **Examples of Health Services and Conditions Not Included in EUHC**

- Routine physical exams
- Low back pain (except some basic physical management)
- Allergies
- Shingles vaccine
- Common viral infections^a^
- Routine urinary tract infection^a^
- Juvenile diabetes
- Gastric reflux and heartburn
- Colonoscopy/mammography
- Prostatic hypertrophy
- Infertility
- Inflammatory bowel disease
- Hemorrhoids
- Cosmetic surgery
- Fibromyalgia
- Lupus
- Multiple sclerosis
- Alzheimer's disease
- Parkinson's disease
- Joint replacement
- Treatment of all cancer except early cervical, breast, and colorectal
- Medium and advanced treatment of most cardiovascular disease

Abbreviation: EUHC, Essential Universal Health Coverage.

^a^ The HPP does include management of febrile illness but is oriented toward severe disease like malaria.

### Most Impact Comes With the HPP, but Even It Requires Substantial Boost in Health Spending

The Figure shows the projected reduction in premature deaths and the annual cost in percent of gross national income (GNI) for the HPP and the EUHC, assuming 80% population coverage at 2030. (The intersectoral interventions were not included in this analysis.) Notice that for both LICs and LMICs, the **HPP would provide 80% of the death reduction but at about half the incremental cost of the EUHC.** The increased costs beyond what countries are currently spending just for the HPP, however, are substantial and would require **more than a doubling of the percent of GNI dedicated to health by 2030**. And the additional cost of the EUHC is even more formidable at about twice that of the HPP. Not surprisingly, the population-based and community platforms had the lowest costs.

**FIGURE:**
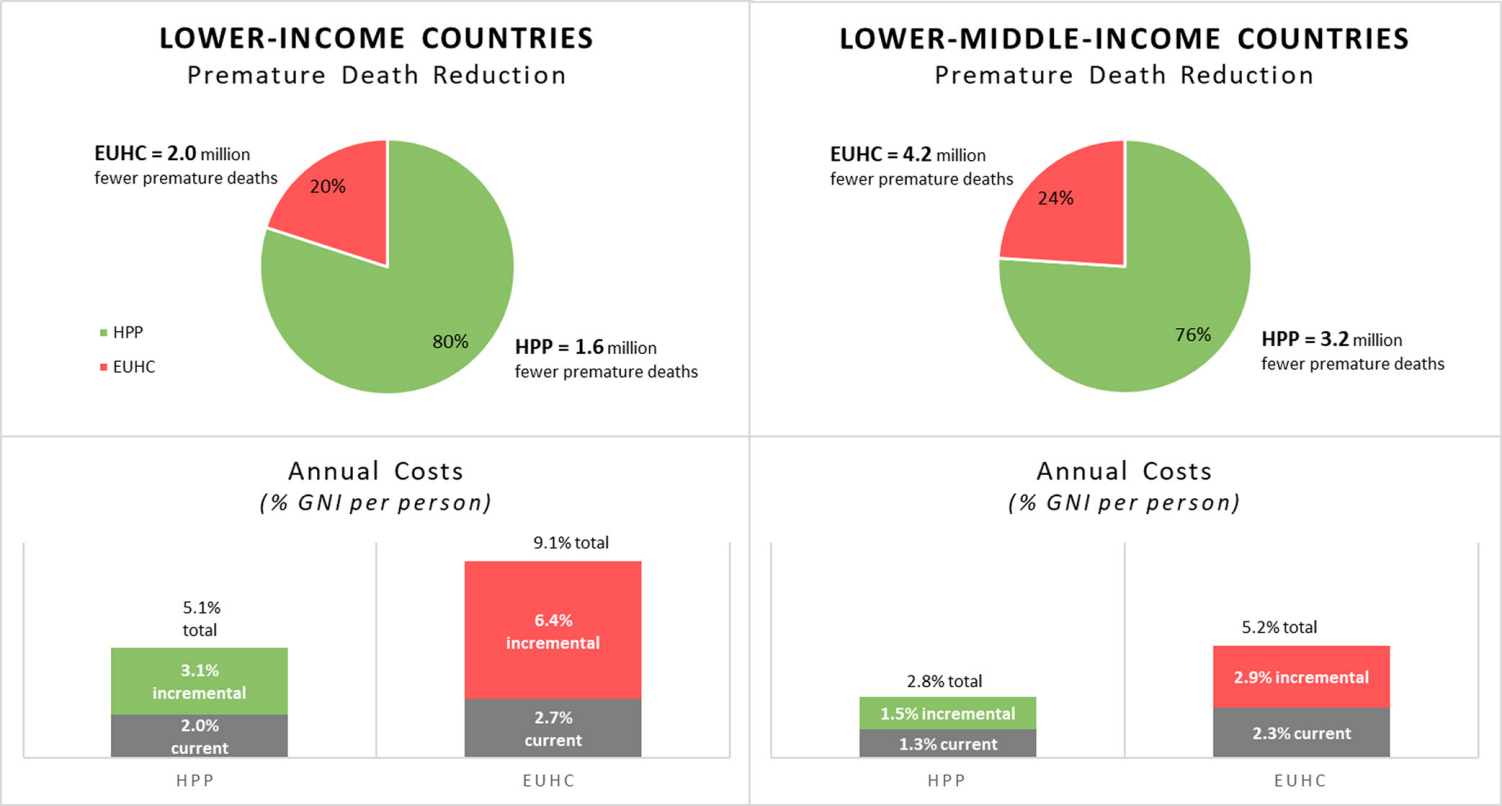
Health Impact and Annual Cost of HPP and EUHC in Lower-Income and Lower-Middle-Income Countries Abbreviations: HPP, Highest Priority Package; EUHC, Essential Universal Health Coverage; GNI, gross national income.

### Implementation of Even the More Expensive EUHC Would Fall Far Short of 2030 SDGs

The SDGs translate into a decline in premature mortality of 40%, or 7.0 million lives, annually by 2030 in LMICs. However, as shown in the Figure, the projected impact of EUHC is a reduction of only 4.2 million lives annually—about 40% short of the objective.

On a more positive note, substantial additional impact could come from implementing a good portion of the intersectoral approaches, which were not included in the impact and cost analysis. And some approaches, like tobacco and alcohol taxes, can actually generate substantial funding. However, others like improved outdoor air quality and safer highway design would come at additional cost. To reach the SDGs, we may also benefit from the tailwind of advancing economic development wherein much of the gains in health indictors historically have come from incompletely understood effects through such means as improved nutrition, sanitation, housing, communication, transportation, and health literacy. On the other hand, economic development can lead to increases in many chronic diseases, which are becoming increasingly important.

### Avoiding Extreme Poverty

Avoiding extreme poverty is an integral UHC objective. Such poverty can come from high out-of-pocket expenditures as well as time lost from work from either sustained illness or a catastrophic episode. Still it is not at all clear that purely subsidizing clinical services, as UHC is too often conceived, is the best remedy for this problem. Rather, a strong emphasis on preventing severe and chronic disease through interventions like tobacco control, clean air, bed nets, and traffic safety, along with some key therapeutic components such as those laid out in DCP3, may well be a better approach.

## FACING THE HARSH REALITY OF ENORMOUS HEALTH NEEDS BUT VERY LIMITED RESOURCES—INFORMED PRIORITIZING IS CRUCIAL

We would all like to live in a world where all health need could be satisfied. Of course, like any overarching exercise of its kind, DCP3 surely entails some inaccuracies and is subject to necessary assumptions and simplifications. Still there is no escaping the conclusion, that health needs across the board vastly outstrip realistic resources. Proper prioritizing is thus imperative.

### Public Health Leadership Should Frame UHC Around Actual Health Impact

Given our current reality where health need vastly outstrips resources, where many decision makers are mostly oriented toward curative services, and where decision making takes place on the uncertain political stage, what should public health leadership do? Serious health advocates should weigh in to make the case for the most cost-effective health interventions, adapted to country context and subject to political realities. That includes advocacy with political leaders and the public for intersectoral health promoting interventions like clean air initiatives, taxes on tobacco and alcohol, and traffic calming as well as clinical services like a full set of childhood immunizations—not just as having access to a health care provider when a person may want or need one. Encouragingly, WHO has recently weighed in advocating a more largely “intersectoral” preventive approach to non-communicable diseases.^7^

Clearly, undertaking UHC under these circumstances is a difficult and uncertain process. No wonder then in the current issue of GSHP, Paul and colleagues find very diverse views from informed health experts about how to go about UHC, even in the fairly homogeneous context of francophone Africa.^8^ That appears to be at least partly because the evidence for how to implement UHC is very weak, even once the decision is made as to what it should consist of. Nevertheless, some serious efforts are underway to try to rationalize the UHC process—for example, the recent volume from the Center for Global Development, *What's In, What's Out: Designing Benefits for Universal Health Coverage*.^9^ But the authors of DCP3 have now given us a concrete, robust evidence-based template to turn the attention of UHC to interventions that will benefit health the most.

## CONCLUSION

The authors of DCP3 have done a great service by identifying, with painstaking effort and expertise, key interventions that will have the most health impact for the universe of people in LMICs. Their priority packages do respond to the felt need for clinical services at the facility level. But the most impactful priority interventions are at the population, community, and intersectoral levels. The rest of the global health community should follow the leadership of DCP3, get beyond the mentally constraining framework around “health services,” and emphasize true health impact in UHC. The people we serve deserve nothing less.

## Notes

**First Published Online:** June 14, 2018
